# Volume change rate before and after neoadjuvant systemic therapy of breast cancer is an efficacious evaluation index to predict pathological complete response

**DOI:** 10.3389/fonc.2023.910869

**Published:** 2023-02-06

**Authors:** Yinggang Xu, Weiwei Zhang, Siqi Wang, Lu Xu, Haiping Xu, Rui Chen, Xiaoqing Shi, Xiaofeng Huang, Ye Wang, Jinzhi He, Wenjie Shi, Xinyu Wan, Jue Wang, Xiaoming Zha

**Affiliations:** ^1^ Department of Breast disease, the First Affiliated Hospital of Nanjing Medical University, Nanjing, China; ^2^ Department of Radiology, the First Affiliated Hospital of Nanjing Medical University, Nanjing, China; ^3^ Collaborative Innovation Center for Cancer Personalized Medicine, Nanjing Medical University, Nanjing, China

**Keywords:** breast cancer, neoadjuvant systematic therapy (NST), anthracycline, taxane, volume measurements

## Abstract

Neoadjuvant systemic therapy (NST) is widely applied in breast cancer treatment, but individuals respond differently to the same NST regimen. It is unclear which patients should adjust their NST regimen and what such an adjustment should be, especially for patients with radiologically partial response (PR). This study aimed to identify a quantitative efficacy evaluation index to evaluate the therapeutic effect of NST. 164 patients were enrolled in this study received four cycles of epirubicin and cyclophosphamide (EC), followed by four cycles of taxanes with trastuzumab [T(H)], if needed. Of patients with a volume change rate of EC treatment (δV1) below 0.80, more than half benefited from subsequent T(H) treatment compared with EC treatment. Importantly, for δV1 of 0.80 and higher, patients’ subsequent T(H) treatment was not as efficient as previous EC treatment and they have a lower pathological complete response (pCR) rate. Across all patients, nanoparticle albumin-bound paclitaxel had a numerically higher pCR rate over other taxanes in patients with triple-negative breast cancer. This study showed that the volume change rate is better than the diameter change rate in monitoring the therapeutic effect of NST. Furthermore, δV1 is a good quantitative efficacy evaluation index to distinguish patients resistant to EC treatment and predict the pCR rate and guide the adjustment of individualized NST regimens.

## Introduction

Breast cancer is the most common malignant tumor in females worldwide (1, 2). Neoadjuvant systemic therapy (NST) is increasingly adopted and widely applied in breast cancer treatment not only for locally advanced breast cancer, but also for early case (3, 4). NST can bring surgical benefit for those who have inoperable disease (5) or who want to preserve the breast.

However, it is worth noting that NST does not bring additional survival benefits (6, 7). The reason may lie in the fact that the difference between neoadjuvant and adjuvant systemic therapy is the sequence of systemic therapy and surgery rather than the systemic therapy regimen itself. Evidence has already shown that an adjusted systemic regimen brings survival benefits for some patients. Patients with triple-negative breast cancer (TNBC) and positive for human epidermal growth factor receptor 2 (HER2) who did not achieve pathological complete response (pCR) can have better survival after escalating adjuvant systemic therapies (8, 9). Similarly, *in vivo* information of therapeutic effect during NST is also useful to guide adjusting NST regimen (10, 11). For example, when evaluated as radiologically progressive disease (PD) or stable disease (SD), previously ineffective agents should be replaced. Nevertheless, for patients with radiologically partial response (PR), since the definition of PR has a wide range, and the degree of individualized agent sensitivity of patients is also different, whether the NST regimen should be adjusted remains unclear. Thus, a quantitative efficacy evaluation index is necessary for NST adjustment.

In our center, we included patients with breast cancer with radiologically measurable primary lesions. During the entire NST treatment process, data of tumor changes were recorded and then quantified into two indexes: diameter change rate (δL) and volume change rate (δV). δL and δV were analyzed respectively and compared for their differences in evaluating the therapeutic effect of NST. The aim of this study was to identify a quantitative efficacy evaluation index to evaluate the therapeutic effect of NST.

## Materials and methods

### Patients

Eligible patients were female with operable invasive breast cancer confirmed by core needle biopsy. Estrogen receptor (ER), progesterone receptor (PgR), HER2, and the nuclear protein Ki67 were evaluated by immunohistochemical (IHC) staining. Patients should have completed all cycles of epirubicin, and cyclophosphamide (EC) followed by taxane (T) regimen. Trastuzumab was concurrently applied with taxane (TH) when anti-HER2 target therapy is necessary. Magnetic resonance imaging (MRI) was used before the first and fifth cycles of NST, as well as before surgery.

The exclusion criteria were as follows: previous chemotherapy or targeted therapy; distant metastatic lesions; severe concomitant diseases such as infection, uncontrolled diabetes, malignant hypertension, or hemorrhagic diseases; peripheral neuropathy; discontinued NST; aspartate aminotransferase and/or alanine aminotransferase 1.5 times higher than the normal upper limit; serum creatinine levels exceeding 1.5 times the normal upper limit; white blood cell count less than 3.5 × 10^9^/L; neutrophil count below 2.0 × 10^9^/L or platelet count less than 90 × 10^9^/L; left ventricular ejection fraction below 55% at baseline; and Eastern Cooperative Oncology Group performance status greater than 1; Unmeasurable tumor without discernible boundary; tumor scattered or discontinuous loci after NST.

### Methods

**Figure 1:**
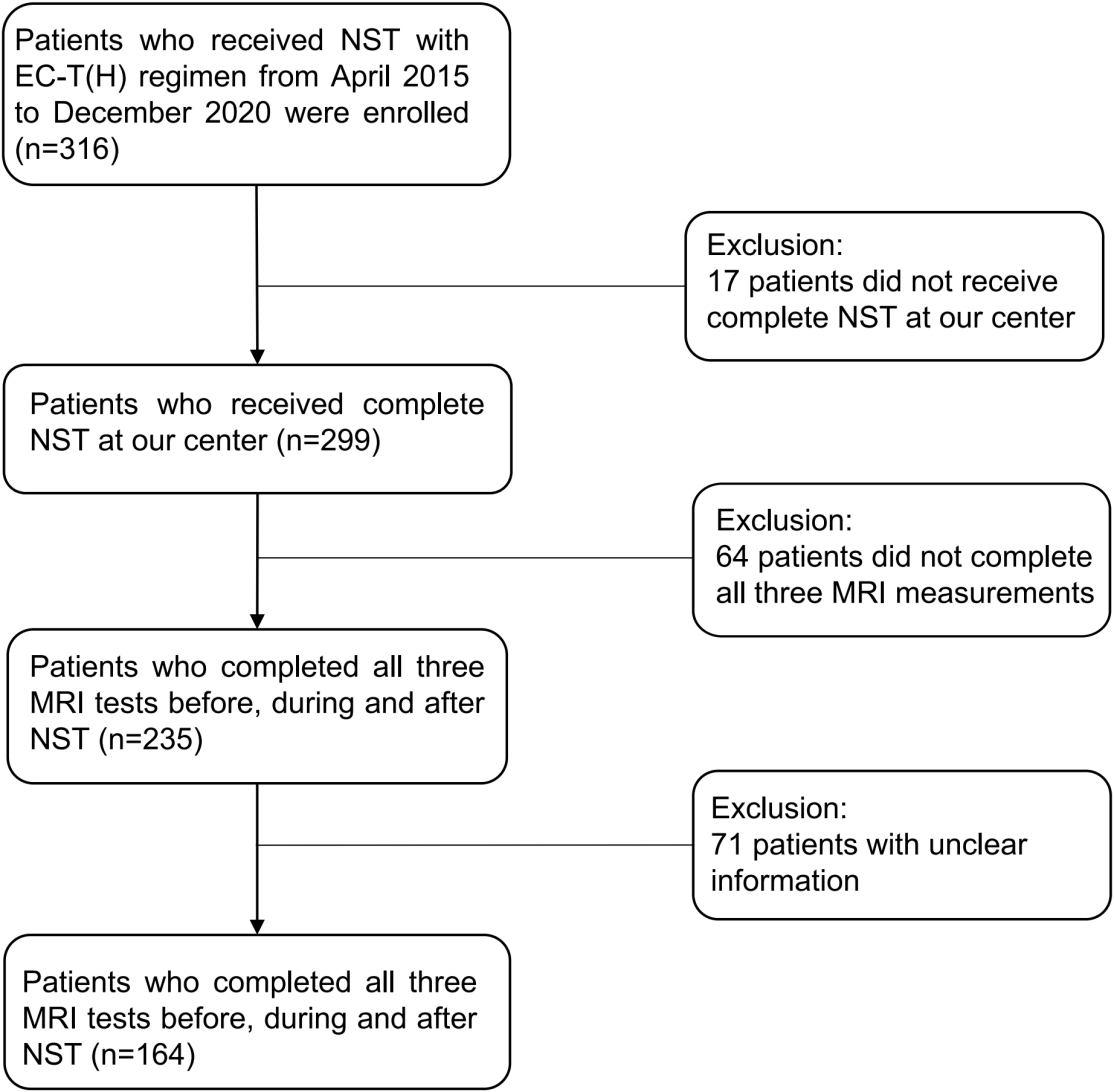
Flow chart of the study design. NST, neoadjuvant systemic treatment; EC-T(H), epirubicin and cyclophosphamide followed by paclitaxel with trastuzumab if needed.

This is a retrospective study conducted at a single breast cancer center. 164 patients patients who were diagnosed with invasive breast cancer and received NST with EC-T were enrolled in this study (**Figure 1**). They have completed 8 NST cycles and 3 MRI tests before, during and after NST at our center.

ER and PgR positivity was defined as more than 1% of positive cells by nuclear staining. HER2 positivity (HER2+) was defined as 3+ on IHC staining or IHC staining 2+ with HER2 gene amplification by fluorescence *in situ* hybridization (FISH). The molecular subtypes of breast cancer were defined according to hormone receptor (HR) and HER2 status.

All patients received four cycles of EC (Pharmorubicin, 90 mg/m^2^, day 1, every 14 or 21 days; and Endoxan, 600 mg/m^2^, day 1, every 14 or 21 days) treatment followed by four cycles of taxane treatment, including nab-P (Abraxane, 260 mg/m^2^, day 1, every 14 days) or sb-P (Taxol, 175 mg/m^2^, day 1, every 14 days) or docetaxel (Taxotere, 75 mg/m^2^, day 1, every 21 days) or liposome (Paclitaxel Liposome for Injection, 175 mg/m^2^, day 1). For HER2+ patients, trastuzumab (Herceptin, 6 mg/kg every 21 days with 8 mg/kg as a loading dose) was used from the fifth cycle with taxanes.

**Figure 2:**
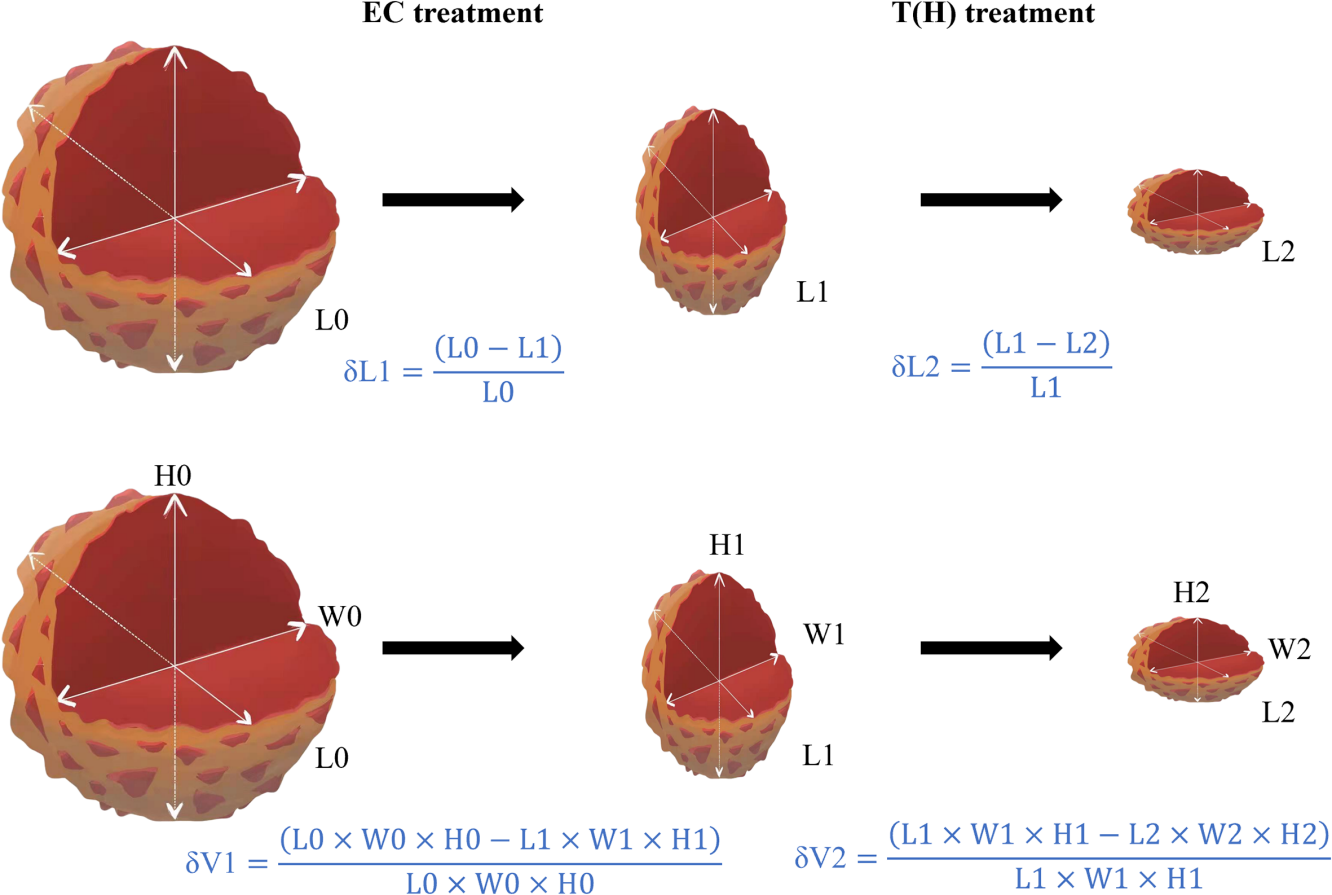
Methods for calculating diameter change rate (δL) and the volume change rate (δV). Abbreviations: EC, epirubicin and cyclophosphamide; T(H), taxanes (Trastuzumab); L, longest diameter; H, height; W, width; δL1, diameter change rate of EC treatment; δL2, diameter change rate of T(H) treatment; δV1, volume change rate of EC treatment; δV2, volume change rate of T(H) treatment.

The complete blood count, liver function, renal function, and electrocardiogram of each patient were monitored before each cycle of NST and before surgery. If the patient had febrile neutropenia, grade 4 neutropenia, grade 4 thrombocytopenia, or grade 4 non-hematological toxicity (except nausea, vomiting, and fatigue), the dosage of the NST regimen was reduced. Surgery was performed about 1 month after completion of NST. Diameters in three directions (length/hight/width) are typically stated in MRI reports, and the maximum of the three reported diameters is most frequently used for clinical evaluation perviously. Response Evaluation Criteria in Solid Tumors (RECIST) sets the standard for maximum diameter in whatever direction, which is the most commonly used measure for breast cancer efficacy monitoring. According to MRI data, δL1 is defined as the maximum diameter change rate of EC treatment, whereas δL2 is calculated for evaluating T(H) treatment. Similarly, δV1 and δV2 are defined as the volume change rate of EC and T(H) treatment, respectively (**Figure 2**). According to Response Evaluation Criteria in Solid Tumors (RECIST) v1.1 (12): CR is defined as the disappearance of all target lesions plus a reduction of the short axis of pathologic lymph nodes to less than 10 mm. PR is defined as at least 30% decrease in the sum of the maximum diameters of target lesions, whereas PD is defined as at least 20% increase (≥5 mm absolute increase) in the sum of the maximum diameters of target lesions or the appearance of new lesions; SD is neither PR nor PD. Pathology was diagnosed by an experienced pathologist. pCR is defined as no invasive tumor residue in breast and no invasive or noninvasive tumor residue in axillary lymph nodes (ypT0/is ypN0).

All procedures were performed in line with the ethical standards of the committees (institutions and countries) responsible for human experiments and the Helsinki Declaration. This study was approved by the Ethics Research Committee of the First Affiliated Hospital of Nanjing Medical University (2021-SR-495). Informed consent of all patients was obtained for inclusion in the study.

### Statistical analysis

Patient and tumor characteristics were compared between groups by Pearson’s χ^2^ test or Fisher’s exact test. IBM SPSS Statistics (IBM Corp., v26.0, Armonk, NY, USA) was used for statistical analysis. The cutoff value of δV1 was calculated using R (version 3.6.1) with the package “cutpointr” and the min(abs(se-sp)) index (13). P < 0.05 was considered statistically different.

## Results

### Patient and tumor characteristics

From May 2015 to Nov 2020, 164 patients were included in this study (mean age: 51.1 ± 10.1 years). The proportion of different molecular subtypes was consistent with the natural distribution of patients with breast cancer. Many patients were cT2 (74.4%), and almost 60% of patients had axillary lymph node involvement. Most patients (92.1%) received modified radical mastectomy. Pathological examination showed that 18.3% of patients achieved pCR (ypT0/is ypN0) (**Table 1**).

**Table 1:** Characteristics of included patients.

Characteristics	No.	%
Included patients	164	
Age (years)	51.1 ± 10.1	
Menstrual status		
Premenopausal	72	43.9
Postmenopausal	92	56.1
Molecular subtypes		
HR+/HER2−	82	50.0
HR+/HER2+	27	16.5
HR−/HER2+	23	14.0
HR−/HER2−	32	19.5
cT at diagnosis		
cT1	8	4.9
cT2	122	74.4
cT3	34	20.7
cN at diagnosis		
cN0	66	40.2
cN+	98	59.8
Surgeries		
Breast-conserving surgery	13	7.9
Modified radical mastectomy	151	92.1
Pathological response to NST		
pCR	30	18.3
pPR	97	59.1
pSD	34	20.7
pPD	3	1.8

HR, hormone receptor; HER2, human epidermal growth factor receptor 2; cT, clinically assessed tumor stage; cN, clinically assessed axillary node stage; NST, neoadjuvant systemic therapy; pCR, pathological complete response, no invasive tumor residue in breast and no invasive or non-invasive tumor residue in axillary lymph nodes (ypT0/is ypN0); pPR, pathological partial response; pSD, pathological stable disease; pPD, pathological progressive disease.

### pCR rate was similar between groups when evaluated by diameter (δL) or volume (δV) change rates

First, δL1 and δL2 were used to evaluate the therapeutic response. δL1 < δL2 means higher relative diameter change for T(H) than EC treatment, while δL1 ≥ δL2 means equal or higher relative diameter change for EC than T(H) treatment. Results showed that about half of the patients (53.7%) were δL1 < δL2, and nearly half of the patients (46.3%) were δL1 ≥ δL2. Although the pCR rate was higher in the δL1 < δL2 group than the δL1 ≥ δL2 group (22.7% vs. 13.2%, respectively), no statistical difference was found. For HER2+ patients, the proportion of those in the δL1 < δL2 group was twice that of the δL1 ≥ δL2 group (66.0% vs. 34.0%. respectively), but the pCR rates were not statistically different (Table 2). For HER2− patients, the proportion of δL1 < δL2 patients (48.2%) were similar to δL1 ≥ δL2 patients (51.8%), and the pCR rates were similar between the two groups(**Table 2**). Next, δV1 and δV2 were used to also evaluate the therapeutic response. Although the specific values are different from δL, the main conclusions of δV were the same as those of δL (**Table 2**).

**Table 2:** pCR rates were similar between groups when evaluated by δL and δV.

		δL				δV		
Molecular subtype	Therapeutic response	Patients No.(%)	pCR No. (%)	P	Therapeutic response	Patients No. (%)	pCR No. (%)	P
**All patients (n = 164)**	δL1 < δL2	88 (53.7)	20 (22.7)	0.114	δV1 < δV2	89 (54.3)	19 (21.3)	0.270
	δL1 ≥ δL2	76 (46.3)	10 (13.2)		δV1 ≥ δV2	75 (45.7)	11 (14.7)	
**HER2+ patients (n = 50)**	δL1 < δL2	33 (66.0)	12 (36.4)	0.357	δV1 < δV2	36 (72.0)	12 (33.3)	1.000
	δL1 ≥ δL2	17 (34.0)	4 (23.5)		δV1 ≥ δV2	14 (28.0)	4 (28.6)	
**HER2− patients (n = 114)**	δL1 < δL2	55 (48.2)	8 (14.5)	0.477	δV1 < δV2	53 (46.5)	7 (13.2)	0.779
	δL1 ≥ δL2	59 (51.8)	6 (10.2)		δV1 ≥ δV2	61 (53.5)	7 (11.5)	

pCR, pathological complete response, no invasive tumor residue in breast and no invasive or non-invasive tumor residue in axillary lymph nodes (ypT0/is ypN0); HER2, human epidermal growth factor receptor 2; δL1, the longest diameter change rate of EC treatment; δL2, the longest diameter change rate of T(H) treatment; δV1, the volume change rate of EC treatment; δV2, the volume change rate of T(H) treatment.

### δV1 was better than δL1 in subgroup analysis of pCR rate

Using the mean value of δL1 (0.33) to divide patients into two groups, a higher pCR rate was observed in the δL1 ≥ 0.33 group than the δL1 < 0.33 group (29.2% vs. 9.8%, respectively, *P* = 0.001). Subgroup analysis showed that in the δL1 < 0.33 subpopulation, no difference in pCR rate was found between the δL1 < δL2 and δL1 ≥ δL2 subgroups. Similarly, in the δL1 ≥ 0.33 subpopulation, no difference in pCR rate was also found between the two subgroups (**Table 3**).

**Table 3:** Comparisons of pCR rates among different δL1 subgroups and δV1 subgroups.

	δL				δV		
Therapeutic response	Patients No. (%)	pCR No. (%)	P	Therapeutic response	Patients No. (%)	pCR No. (%)	P
**All patients**	164 (100)	30 (18.3)		**All patients**	164 (100)	30 (18.3)	
**δL1 < 0.33**	92 (56.1)	9 (9.8)	0.001^a^	**δV1 < 0.66**	78 (47.6)	7 (9.0)	0.003^b^
δL1 < δL2 subgroup	55 (59.8)	7 (12.7)	0.423	δV1 < δV2 subgroup	54 (69.2)	5 (9.3)	1.000
δL1 ≥ δL2 subgroup	37 (40.2)	2 (5.4)		δV1 ≥ δV2 subgroup	24 (30.8)	2 (8.3)	
**δL1 ≥ 0.33**	72 (43.9)	21 (29.2)		**δV1 ≥ 0.66**	86 (52.4)	23 (26.7)	
δL1 < δL2 subgroup	33 (45.8)	13 (39.4)	0.079	δV1 < δV2 subgroup	35 (40.7)	14 (40.0)	0.021^c^
δL1 ≥ δL2 subgroup	39 (54.2)	8 (20.5)		δV1 ≥ δV2 subgroup	51 (59.3)	9 (17.6)	

pCR, pathological complete response, no invasive tumor residue in breast and no invasive or non-invasive tumor residue in axillary lymph nodes (ypT0/is ypN0); HER2, human epidermal growth factor receptor 2; 0.33, the mean value of δL1; 0.66, the mean value of δV1; δL1, the longest diameter change rate of EC treatment; δL2, the longest diameter change rate of T(H) treatment; δV1, the volume change rate of EC treatment; δV2, the volume change rate of T(H) treatment; ^a^, pCR rate compared with the δL1 ≥ 0.33 group; ^b^, pCR rate compared with the δV1 ≥ 0.66 group; ^c^, pCR rate compared with the δV1 ≥ δV2 subgroup; ^d^, pCR rate compared with the δV1 ≥ δV2 subgroup.

Using 0.66, which was the mean value of δV1 in our cohort, as a cutoff value, a higher pCR rate was also observed in the δV1 ≥ 0.66 group than the δV1 < 0.66 group (26.7% vs. 9.0%, respectively, *P* = 0.003). Subgroup analysis showed no differences in the pCR rate between the δV1 < δV2 and δV1 ≥ δV2 subgroups in the δV1 < 0.66 subpopulation. However, in the δV1 ≥ 0.66 subpopulation, the δV1 < δV2 subgroup demonstrated a significantly higher pCR rate than the δV1 ≥ δV2 subgroup (40.0% vs. 17.6%, respectively, *P* = 0.021) (**Table 3**).

**Figure 3:**
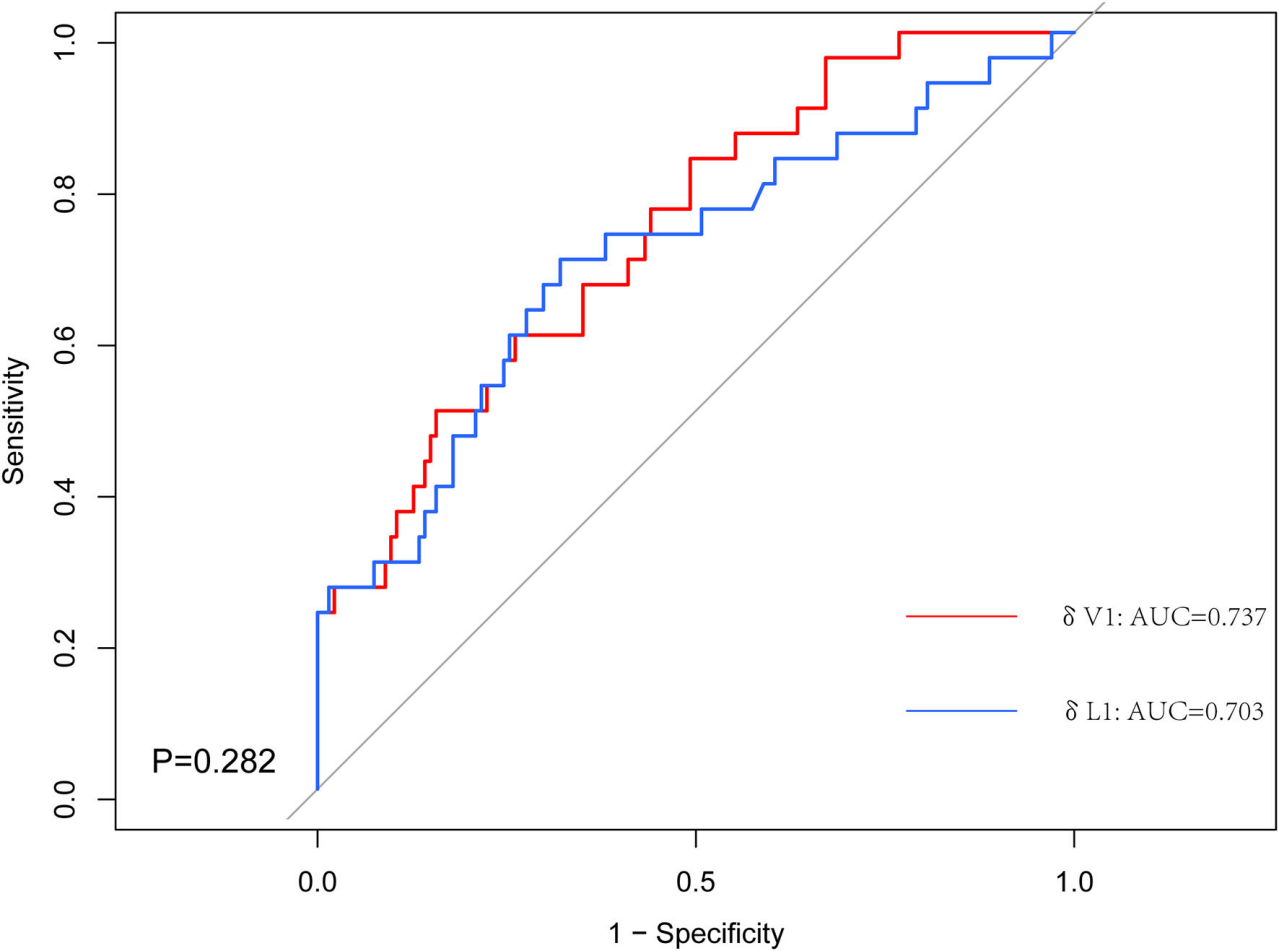
The receiver operating characteristic (ROC) curves for the δV1 and δL1. The area under the ROC curve (AUC) for δV1 was significantly higher.

**Figure 4:**
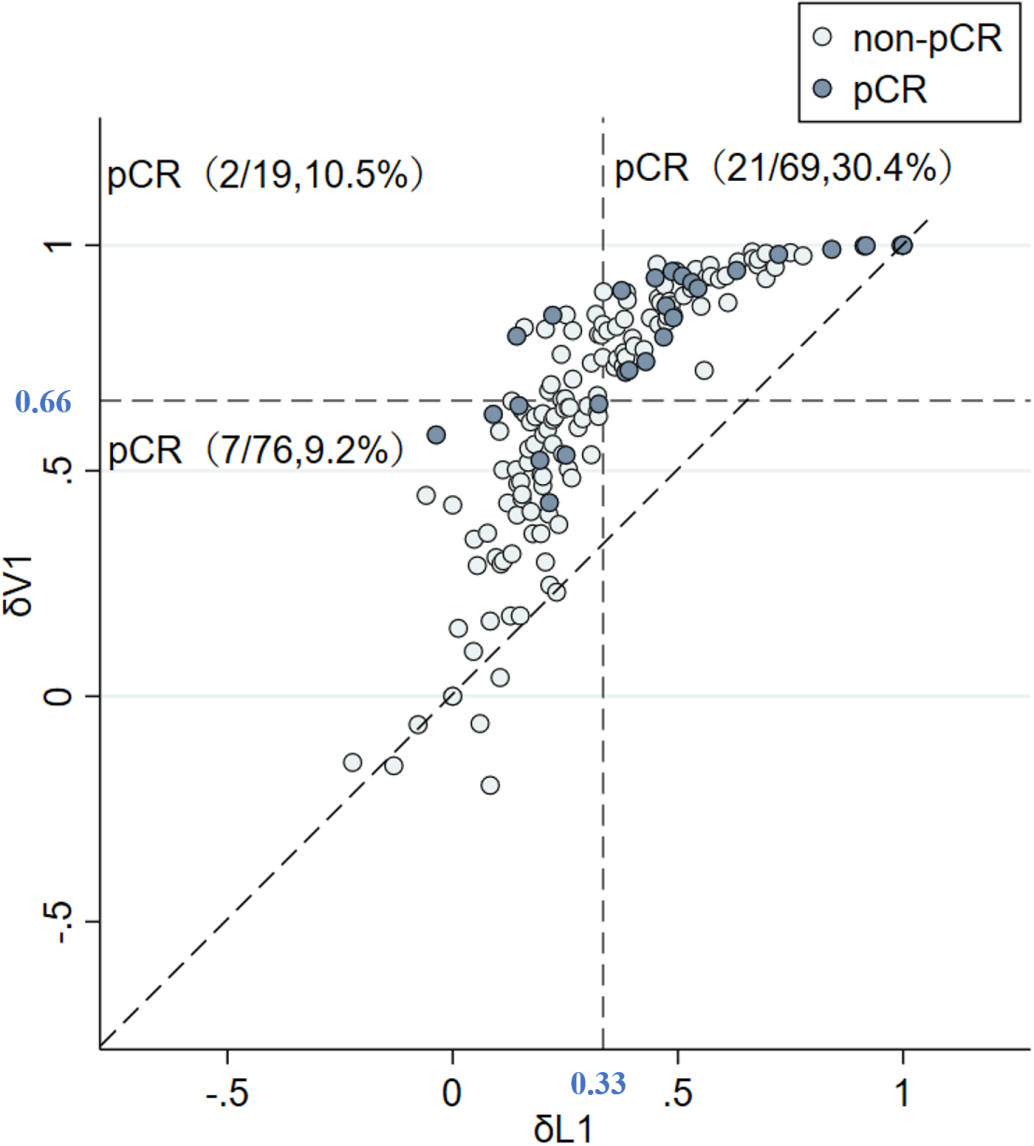
δV1 is better than δL1 in evaluating tumor change rate. δV1 did not underestimate the effective cases judged by δL1, and it also identified underestimated cases by δL1. Abbreviations: pCR, pathological complete response; non-pCR, non-pathological complete response; δV1, volume change rate of EC treatment; δL1, longest diameter change rate of EC treatment.

The predictive capability was assessed using the area under the curve (AUC). The AUC index of δV1 was 0.737, and the AUC index of δL1 was 0.703 (**Figure 3**). According to measures of δL1 and δV1, patients were compared case by case (**Figure 4**), and the results showed that most cases were above the 45° dashed line. The pCR rate in the left lower, left upper, and right upper quadrant was 9.2%, 10.5%, and 30.4%, respectively. No patient was located in the right lower quadrant.

### Higher pCR rate was observed in the δV1 < δV2 subgroup of the δV1 ≥ 0.80 subpopulation

The min(abs(se-sp)) index of 0.80 (exact value 0.7985) was used as a threshold for further analysis of the δV1 ≥ 0.66 subpopulation (Additional file 1: **Figure S1**). In the 0.66 ≤ δV1 < 0.80 subpopulation, more than half of the patients were δV1 < δV2, and nearly half of them were δV1 ≥ δV2. No statistical difference in pCR rate was found between the δV1 < δV2 and δV1 ≥ δV2 subgroups. In comparison, the probability that δV1 is less than δV2 was only about 1/3, but the pCR rate of the δV1 < δV2 subgroup was significantly higher than the δV1 ≥ δV2 subgroup (43.5% vs. 19.0%, respectively, *P* = 0.035) (**Table 4**).

**Table 4:** Higher pCR rates were observed in the δV1 < δV2 subgroup of the δV1 ≥ 0.80 subpopulation.

Therapeutic response	Patients No. (%)	pCR No. (%)	*P*
**0.66 ≤ δV1 < 0.80**	21	5 (23.8)	0.727
δV1 < δV2 subgroup	12 (57.1)	4 (33.3)	0.338
δV1 ≥ δV2 subgroup	9 (42.9)	1 (11.1)	
**δV1 ≥ 0.80**	65	18 (27.7)	
δV1 < δV2 subgroup	23 (35.4)	10 (43.5)	0.035^a^
δV1 ≥ δV2 subgroup	42 (64.6)	8 (19.0)	

pCR, pathological complete response, no invasive tumor residue in breast and no invasive or non-invasive tumor residue in axillary lymph nodes (ypT0/is ypN0); 0.66, the mean value of δV1; 0.8, 0.80, the threshold value of δV1; δV1, the volume change rate of EC treatment; δV2, the volume change rate of T(H) treatment; ^a^, pCR rate compared with the δV1 ≥ δV2 subgroup.

### pCR rates were similar among different taxanes

Treatment with different taxanes resulted in statistically similar pCR rates in our study cohort. In addition, no significant difference in pCR rate was found among the different molecular patient subgroups for each type of taxane. For HR−/HER2− patients, nab-P treatment showed a high pCR rate of 50.0% (7/14). Although the value was much higher than other taxanes, no statistical difference was found due to the limited number of patients (**Table 5**).

**Table 5:** pCR rates were similar among different taxanes.

Group	nab-P No. (%)	sb-P No. (%)	Docetaxel No. (%)	Liposome No. (%)	P
**All patients**	13/55 (23.6)	6/42 (14.3)	7/47 (14.9)	4/20 (20.0)	0.597
HR+/HER2−	3/25 (12.0)	0/19 (0.0)	1/31 (3.2)	0/7 (0.0)	0.353
HR+/HER2+	1/10 (10.0)	1/8 (12.5)	2/7 (28.6)	1/2 (50.0)	0.481
HR−/HER2+	2/6 (33.3)	4/8 (50.0)	3/4 (75.0)	2/5 (40.0)	0.659
HR−/HER2−	7/14 (50.0)	1/7 (14.3)	1/5 (20.0)	1/6 (16.7)	0.337

pCR, pathological complete response, no invasive tumor residue in breast and no invasive or non-invasive tumor residue in axillary lymph nodes (ypT0/is ypN0); HR, hormone receptor; HER2, human epidermal growth factor receptor 2; δV1, the volume change rate of EC treatment; δV2, the volume change rate of T(H) treatment; nab-P, nanoparticle albumin-bound paclitaxel; sb-P, solvent-based paclitaxel; Liposome, Liposome paclitaxel for Injection.

## Discussion

Anthracycline agents are the cornerstone of breast cancer chemotherapy (14), whereas the addition of sequential taxanes to preoperative anthracycline-containing regimens can significantly increase the pCR rate of operable breast cancer (15, 16). Moreover, anti-HER2 agents are also needed for NST of HER2+ tumors (17). Therefore, EC followed by a T(H) regimen is used for NST in our center. In this study, 164 patients were included and half of them (50.0%) belonged to the HR+/HER2− subtype, whereas HER2+ patients accounted for 30.5% and HR−/HER2− (triple-negative) patients accounted for 19.5% of our cohort. This distribution of the enrolled subgroup population is similar to the real world.

In the process of clinical treatment, patients respond differently to the same NST regimen. This difference manifests not only among different patients but also between EC and T(H) treatments of the same patient. To compare the therapeutic effects between EC and T(H) in our study, the maximum diameters of the primary lesion were regularly monitored and evaluated. δL1 is defined as the maximum diameter change rate of EC treatment, while δL2 is calculated to evaluate T(H) treatment. Further, since the maximum diameter of the primary lesion can shrink a little but the diameters of other lesions shrink a lot in some cases, the volume change rate (δV) was also calculated. Similarly, δV1 is defined as the volume change rate of EC treatment, whereas δV2 is defined for T(H) treatment.

Results showed that patient population between δL1 < δL2 (δV1 < δV2) and δL1 ≥ δL2 (δV1 ≥ δV2) were similar, indicating that both EC and T(H) are effective during NST. However, the pCR rate of the δL1 < δL2 (δV1 < δV2) group was higher than that of the δL1 ≥ δL2 (δV1 ≥ δV2) group. This difference was attributed to the HER2+ subgroup because about 2/3 or 3/4 HER2+ subgroup patients showed a higher proportion in the δL1 < δL2 or δV1 < δV2 groups, respectively, than the δL1 ≥ δL2 and δV1 ≥ δV2 groups, consequently resulting in a higher pCR rate in these HER2+ subgroups (**Table 2**). It is reasonable that targeted therapy can enhance the anti-tumor effect on HER2+ breast cancer (17, 18).

Considering irreversible cardiotoxicity of anthracycline agents, such as dilated cardiomyopathy and supraventricular tachycardia, research on anthracycline-free regimens is a current hot topic (19–24). However, two randomized trials have shown that for breast cancer patients with triple-negative or axillary lymph node metastasis, anthracycline agents still provide significant therapeutic benefits (25, 26). According to our results, the population of δL1 < δL2 (δV1 < δV2) compared with δL1 ≥ δL2 (δV1 ≥ δV2) in the HER2− subgroup were similar, and the pCR rates between these two subgroups were also similar, indicating that both EC and T treatments were equally effective for HER2− patients. However, although targeted therapy is very effective for HER2+ breast cancer, EC treatment achieved a better therapeutic effect than TH regimen (δV1 ≥ δV2) in approximately 25% to 33% of HER2+ patients (**Table 2**). In addition, there are patients who are primarily resistant to trastuzumab and pertuzumab (27). Therefore, the continued role of EC treatment is not to be ignored. Despite these toxicities, there is no anthracycline-free chemotherapy regimen that is superior to an anthracycline-containing regimen for high-risk patients (20). How to screen these patients and provide individualized NST is worthy of further study.

To explore the therapeutic effect of EC treatment, patients were divided into two groups by average values of δL1 (0.33) and δV1 (0.66) and evaluated separately. We found that the pCR rate was low (9.8%) for δL1 < 0.33. In comparison, for δL1 ≥ 0.33, the pCR rate was significantly higher (29.2%, *P* = 0.001). Similarly, using a δV1 of 0.66 as the threshold for analysis, a significant difference in pCR rates was also observed (9.0% vs. 26.7%, respectively, *P* = 0.003) (**Table 3**). These findings indicate that both diameter and volume change rates of EC treatment are predictive of the probability of the pCR rate.

Further, two points are worth noting from the subgroup analysis. First, in δL1 < 0.33 populations, the pCR rate of the δL1 < δL2 subgroup was numerical twice that of the δL1 ≥ δL2 subgroup (12.7% vs. 5.4%, respectively). However, when compared with the volume change rate, the pCR rate of the δV1 < δV2 subgroup was similar to the δV1 ≥ δV2 subgroup (9.3% vs. 8.3%, respectively) (**Table 3**). Second, in δL1 ≥ 0.33 populations, we found that the pCR rate of the δL1 < δL2 subgroup was twice that of the δL1 ≥ δL2 subgroup (39.4% vs. 20.5%, respectively), although the difference was not significantly different (*P* = 0.079) (**Table 3**). In comparison, using measures of δV1, the pCR rate of the δV1 < δV2 subgroup was significantly higher than that of the δV1 ≥ δV2 subgroup (40.0% vs. 17.6%, respectively, *P* = 0.021) (**Table 3**). To explore differences between diameter and volume change rates, the ROC curve was plotted (Fig 3). The AUC index of δV1 was higher than that of δL1, indicating that δV1 may be better in predicting pCR rate than δL1, though the p-value was was not statistically significant (*P* = 0.282). To further analyze the difference between diameter and volume change rates, a scatter plot was plotted. As shown in **Figure 4**, no case was located in the right lower quadrant, reflecting the fact that no patient determined to have a better therapeutic effect by δL1 was misjudged as less effective by δV1. In other words, δV1 did not lower the estimate of any effective case. However, 20.0% of patients (19/95) were regarded as having a lower therapeutic effect by δL1 but were evaluated as having a higher effect by δV1. Among these patients, 10.5% (2/19) achieved pCR. This finding is in concordance with the clinical fact in some patients that the maximum diameter of the tumor changes little during NST, but the other two diameters of the tumor varies greatly. These patients are easily to be underestimated by δL1, but can be accurately assessed by δV1, which is the possible reason for the difference in AUC index between them. A patient proportion of 20.0% is not low, so δV1 may be more suitable for effect evaluation. In addition, a 45° dashed line was used to compare δL1 and δV1. Our results showed that most of the points were above the line, indicating that δV1 can reflect the therapeutic effect more comprehensively than δL1. As a result, δV1 was then used for further calculations and evaluations.

According to our earlier result, for patients whose δV1 was less than 0.66, the pCR rate was 9.0%, which was significantly lower than that of the δV1 ≥ 0.66 subpopulation (*P* = 0.003). This finding is important because it indicates that δV1 can be used as a quantitative index to predict the lower therapeutic effect of EC treatment, and for these patients, EC treatment should be replaced as early as possible. The min(abs(se-sp)) index of 0.80 (0.7985) was used as another threshold for further analysis of the δV1 ≥ 0.66 subpopulation. In the 0.66 ≤ δV1 < 0.80 subpopulation, patients of the δV1 < δV2 and δV1 ≥ δV2 subgroups accounted for about half of each subgroup, indicating that the therapeutic effect of sequential T(H) treatment was comparable to EC regimen. If the therapeutic effect of T(H) treatment is superior to EC treatment, a higher pCR rate can be expected (33.3%, **Table 4**), and as a result, choosing a more appropriate taxane may bring more benefits. Patients in the study were grouped by different taxanes, including nab-P, sb-P, docetaxel, and liposome. Previous studies have reported that nab-P demonstrated a better pCR rate, especially in HR−/HER2− subgroups (28–31). However, due to limited data, no statistical difference was found among different taxanes in the present study. Even in the HR−/HER2− subgroup, there were no statistically significant increases in the pCR rate among different taxane treatments (**Table 5**). In addition, the results in this study showed that the pCR rate of HR+/HER2− lesions was lower than other molecular subgroups, a finding which is consistent with previous trials (i.e., ETNA (32) and GeparSepto trials (28, 29). However, the GeparSepto study proved a survival benefit of nab-P in HR−/HER2− patients (GeparSepto trials (28, 29). This evidence indicates that nab-P is worth trying as a sequential agent. In terms of targeted therapy, only trastuzumab was administered for HER2+ disease in this study, but recent trials recommended trastuzumab and pertuzumab for NST and achieved a better benefit (33, 34). Based on these results, when considering a sequential regimen, taxane plus dual targeted therapy is a better choice.

In the present study, when δV1 was not less than 0.80, the sequential administration of T(H) treatment resulted in a higher (>60%) possibility of less effective than previous EC treatment, which subsequently results in a statistically lower pCR rate (19.0%, *P* = 0.035) (**Table 4**). For these patients, extending the EC treatment may be a theoretically feasible option. Evidence has already shown that four cycles of EC can only achieve results equivalent to CMF regimen, but anthracycline-based regimens with substantially higher cumulative dosages than standard 4EC (e.g., CAF or CEF) bring more survival benefits (35). Furthermore, the dosage of anthracycline agents can be as high as 900–1,000 mg/m^2^ (21, 36) in most patients. Therefore, six or even more cycles of EC are worth trying in anthracycline-sensitive patients under the assessment of cardiac function. Besides, liposomal Adriamycin may also be considered to reduce cardiac toxicity (37).

This study had some limitations. First, it was a retrospective study conducted at a single breast cancer center. As the number of patients enrolled in the study was modest, our results are preliminary, and the detail and depth of subgroup analysis was limited. Second, considering the accuracy of MRI measurements, only patients with measurable and concentric shrinkage lesions were enrolled, which affects the universality of the study’s results and conclusions. Third, because pertuzumab was unavailable in past years, only trastuzumab was administered for HER2+ patients. Moreover, patient tumor burdens were also relatively heavier, leading to a lower pCR rate in this study. Finally, data were collected every four treatment cycles, so it was too late to adjust the regimen of NST. In the future, more frequent monitoring is needed to evaluate the volume change rate (such as once every two cycles), thereby ensuring timely adjustment of the NST regimen.

## Conclusion

This study showed that the volume change rate (δV) is better than the maximum diameter change rate (δL) in monitoring the therapeutic effect of NST. δV1 is a good quantitative efficacy evaluation index to distinguish patients with breast cancer resistant to EC treatment as well as predict the pCR rate, which may help to guide the adjustment of individualized NST regimens.

## Data availability statement

The raw data supporting the conclusions of this article will be made available by the authors, without undue reservation.

## Ethics statement

This study was performed in line with the principles of the Declaration of Helsinki. Approval was granted by the Ethics Committee of the First Affiliated Hospital of Nanjing Medical University (2021-SR-495). The patients/participants provided their written informed consent to participate in this study.

## Author contributions

The authors’ contributions were as follows: JW, andXZ conceptualized and designed the study. All authors completed the acquisition, analysis, and interpretation of the data. JW, HX and XZ obtained the study funding. YX, WZ and JW were responsible for the methodology. JW and XZ provided study supervision. YX drafted the original version of the manuscript. All authors critically revised drafts of the manuscript and approved the final version.
