# Supplementary material for: Volume change rate before and after neoadjuvant systemic therapy of breast cancer is an efficacious evaluation index to predict pathological complete response
Source: Front Oncol. 2023 Feb 6;13:910869. doi: 10.3389/fonc.2023.910869 (PMC9939658; doi:10.3389/fonc.2023.910869)
Supplement: Supplementary file 1 [file DataSheet_1.docx]

**Fig S1**. **The cutoff value of δV1.**

The min (abs(se-sp)) index 0.80 (exact value 0.7985) was used as a threshold for further analysis in δV1≥0.66 subpopulation. The cutoff value was calculated by software R (version 3.6.1) with the package “cutpointr”^[1]^. Min(abs(se-sp)) index, the minimum value of the absolute value of sensitivity minus specificity.


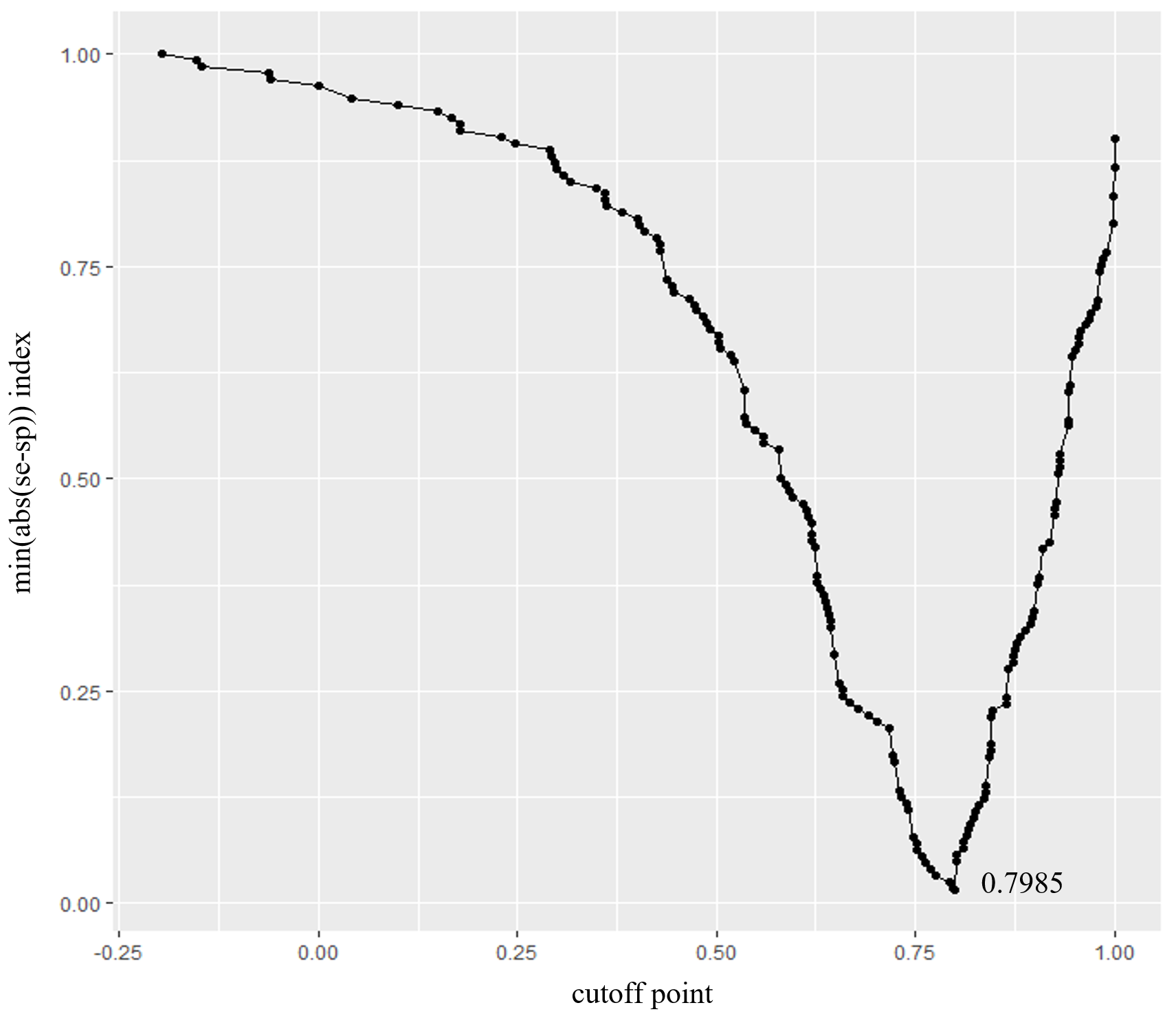


Min, minimum value; abs, absolute value; se, sensitivity; sp, specificity; δV1, the volume change rate of EC treatment.

Reference

1. Thiele C, Hirschfeld G. cutpointr: Improved Estimation and Validation of Optimal Cutpoints in R. 2021 Jun, 98(11).
